# Post-Heart Transplant Changes and Perceived Donor-Recipient Correspondences: A Scoping Review

**DOI:** 10.3390/jcm15176584

**Published:** 2026-08-26

**Authors:** Daniela Nigrelli, Marika Lo Monaco, Mariachiara Figura, Maria Rita Giammarinaro, Irene Zerilli, Israr Ahmad, Laura Iacorossi, Roberto Latina, Giuliano Anastasi

**Affiliations:** 1 Department of Biomedicine and Prevention, University of Rome Tor Vergata, 00133 Rome, Italy; daniela.nigrelli@hotmail.com (D.N.); irenezerilli@hotmail.it (I.Z.); israrahmadd008@gmail.com (I.A.); 2 Department of Maternal and Child Health Promotion, Internal Medicine & Specialties of Excellence “G. D’Alessandro” (PROMISE), University of Palermo, 90127 Palermo, Italy; marika.lomonaco@unipa.it (M.L.M.); roberto.latina@unipa.it (R.L.); 3 Department of Nursing, ARNAS Ospedale Civico di Cristina e Benefratelli, 90127 Palermo, Italy; mariapiagiammarinaro@gmail.com; 4 Department of Life, Health Sciences and Health Professions, Link Campus University, 00165 Rome, Italy; l.iacorossi@unilink.it; 5 Department of Medicine and Surgery, University of Enna “Kore”, 94100 Enna, Italy; giuliano.anastasi@unikore.it

**Keywords:** heart, transplantation, recipients, donors, adaptation, emotional adjustment, self-concept, emotions, nursing, review

## Abstract

**Background/Objectives**: Heart transplantation is a life-saving treatment for advanced cardiac disease, but post-transplant adaptation may involve psychosocial, identity-related, and existential change. Some recipients interpret these changes as corresponding to donor characteristics, although the nature and evidence base of these reports remain unclear. This review mapped post-transplant changes reported by adult heart transplant recipients, perceived donor–recipient correspondences, and the explanatory frameworks proposed for these phenomena. **Methods**: A scoping review was conducted following the Arksey and O’Malley framework, Joanna Briggs Institute guidance, and PRISMA-ScR reporting standards. PubMed, Web of Science, Scopus, CINAHL, and PsychINFO were searched in August 2026. Quantitative, qualitative, mixed-methods, conceptual, opinion, and review articles were eligible. Findings were synthesized descriptively. **Results**: Sixteen sources published between 1992 and 2025 were included. Empirical studies reported changes in preferences, emotional and personality-related experiences, social and physical functioning, sexuality, identity and embodiment, sensory and memory-like experiences, and spirituality. Some changes were attributed by recipients to donor characteristics, whereas correspondences supported by independently obtained donor information were reported in a limited subset of studies. Proposed explanations ranged from psychological, pharmacological, physiological, and sociocultural processes to cellular, molecular, neurocardiac, energetic, and transpersonal hypotheses. Donor-transfer hypotheses were developed in the secondary literature and were not empirically established. Methodological quality varied substantially across sources. **Conclusions**: Post-transplant changes and perceived donor–recipient correspondences are meaningful phenomena, but current evidence does not support the transfer of donor memory, personality, or identity through the transplanted heart. Future prospective, longitudinal, and methodologically rigorous studies should distinguish reported experience, donor attribution, and proposed explanations.

## 1. Introduction

Heart transplantation (HT) has substantially improved survival and health-related quality of life among individuals with advanced cardiac disease [1]. According to data from the International Society for Heart and Lung Transplantation, more than 112,000 heart transplants were performed worldwide between 1992 and 2024, highlighting the role and relevance of HT as a life-saving intervention [2].

Despite these clinical advances, recovery following HT represents a complex psychosocial and biographical transition [3,4,5]. During the post-transplant period, recipients may experience considerable psychological distress. Reported prevalence estimates for stress and anxiety are 21.6% and 11.1%, respectively [6], and recipients may also be at risk of major depressive disorder, generalized anxiety disorder, and post-traumatic stress disorder [7]. Depression, demoralization, pain, and sexual dysfunction have also been reported and may adversely affect the recipients’ quality of life [8]. These difficulties are clinically relevant because they may influence treatment adherence, interpersonal functioning, long-term adaptation, and the need for ongoing psychosocial support [6,9].

Receiving a donor heart also represents an emotionally charged experience in which personal survival is closely associated with another person’s death [10]. This tension may reshape recipients’ identities, priorities, relationships, and processes of meaning-making [11,12]. Qualitative and mixed-methods studies have described changes in emotional responsiveness, self-perception, relational patterns, personal values, and embodied experience following HT [10,11,13]. Some recipients perceive these changes as unexpected, unfamiliar, or difficult to reconcile with their pre-transplant sense of self [10,13,14].

In some studies, recipients have interpreted post-transplant changes as corresponding to characteristics attributed to the donor [14]. To account for such narratives, some authors have drawn on concepts such as cellular or epigenetic memory [15,16,17] and proposed biological or extracerebral mechanisms through which donor-related information might influence recipients after transplantation [18]. However, these explanatory models remain theoretical and are informed by a combination of biological hypotheses, psychological interpretations, and cultural or symbolic frameworks [19].

The available literature remains fragmented across study designs, geographical and cultural contexts, source types, and exploratory perspectives. This fragmentation limits the development of a coherent understanding of how post-transplant changes are experienced and interpreted, how perceived donor–recipient correspondences have been identified, and how these narratives may be addressed in clinical practice. Previous evidence syntheses have examined recipients’ psychological experiences [20], donor and donation imagery [21], the lived experience of heart [10] or thoracic-organ transplantation [13], and neurocognitive or memory-related outcomes [22]. However, to the best of our knowledge, no previous review has simultaneously mapped the range of reported post-HT changes, distinguished different levels of perceived donor–recipient correspondences, and examined the explanatory frameworks proposed across the empirical and non-empirical literature.

Accordingly, this scoping review aimed to map the available evidence on post-transplant changes reported by adult HT recipients and their perceived relationship with donor characteristics. It further examined how donor–recipient correspondences have been assessed or corroborated and mapped the explanatory frameworks proposed to account for post-transplant changes and perceived donor–recipient correspondences.

The following research questions guided the review: (a) What post-transplant changes have been reported by HT recipients? (b) Which post-transplant changes have been perceived as corresponding to donor characteristics, and how have these donor–recipient correspondences been assessed or corroborated? (c) What explanations have been proposed for post-transplant changes and donor–recipient correspondences?

## 2. Materials and Methods

### 2.1. Study Design

A scoping review was conducted, adhering to the methodological framework proposed by Arksey and O’Malley [23] and guidance from the Joanna Briggs Institute (JBI) for conducting scoping reviews [24,25]. The review protocol was not prospectively registered. Reporting followed the Preferred Reporting Items for Systematic Reviews and Meta-Analyses Extension for Scoping Reviews (PRISMA-ScR) [26]. The completed PRISMA-ScR checklist is provided in the Supplementary Materials.

### 2.2. Eligibility Criteria

Eligible sources included primary quantitative, qualitative, and mixed-methods studies involving adult recipients who underwent HT in adulthood and reporting post-transplant changes relevant to behavior, preferences, personality or emotional characteristics, identity or self-concept, bodily or graft-related perceptions, sensory or memory-like experiences, or spiritual dimensions. Studies reporting perceived, assessed, or corroborated correspondences between recipient changes and donor characteristics were also eligible. Reviews, conceptual or theoretical papers, and opinion articles were included when they discussed donor–recipient correspondences or proposed explanatory mechanisms.

Studies were excluded if they focused on psychiatric, neurological, or personality disorders; general adaptation to life after transplantation; caregiver, partner, or family outcomes; waiting-list experiences; or heart failure without transplantation. Studies involving pediatric transplantation, including recipients transplanted during childhood but assessed in adulthood, were excluded. Studies focused on other transplanted organs, left ventricular assist devices (LVADs), or artificial hearts were excluded unless findings for adult HT recipients were separately extractable. Biological, surgical, immunological, and physiological outcomes were outside the scope of the review. Study protocols, instrument-validation studies, books and book chapters, and conference papers or abstracts were also excluded. No restrictions were applied by publication date or language.

### 2.3. Information Sources

A comprehensive literature search was conducted on 10 August 2026 across five databases: PubMed, Web of Science, Scopus, CINAHL Ultimate via EBSCOhost, and PsycINFO via EBSCOhost. All identified records were imported into EndNote version 20.2.1 (Clarivate, Philadelphia, PA, USA) for reference management and deduplication. Duplicate records were first identified using the software’s automated duplicate-detection function and subsequently reviewed manually to identify additional duplicates arising from minor variations in bibliographic information.

### 2.4. Search Strategy

The search strategy combined controlled vocabulary (e.g., Medical Subject Headings [MeSH]), where available, with free-text terms and Boolean operators. Search strings were adapted to the indexing systems and technical requirements of each database.

Three conceptual components informed the search: (1) heart transplantation; (2) post-transplant changes; and (3) donor–recipient correspondences, including terms related to donor-associated traits, memory or personality transfer, cellular memory, and heart memory. These three concepts were operationalized as two search blocks: heart transplantation constituted the first block, while terms relating to post-transplant changes and donor–recipient correspondences were combined with OR to form the second block. The two blocks were then combined using AND.

In accordance with the methodology [24,25,26], the full search strategies for each database are provided in Table A1.

### 2.5. Selection of Sources of Evidence

Source selection was conducted in two stages. First, two reviewers (DN and MRG) independently screened the titles and abstracts of all identified records against the eligibility criteria. Records unrelated to adult heart transplantation, post-transplant changes, donor–recipient correspondences, or proposed explanatory mechanisms were excluded at this stage. Second, two reviewers (RL and MLM) independently assessed the full texts of potentially eligible sources. Backward reference-list screening and forward citation tracking were also performed on full texts to identify additional potentially eligible records. Eligibility was determined according to the predefined population, concept, and source type criteria. For sources including recipients of multiple organ types, inclusion required that findings for HT recipients were separately identifiable and extractable. Any disagreement was resolved through discussion, and when consensus could not be reached, through consultation with a third reviewer (GA).

### 2.6. Data Charting Process

A standardized data-charting form was developed in Microsoft Excel following methodology [24,25]. Two authors (IA and IZ) independently extracted data from each included source. The completed forms were compared, and discrepancies were resolved through discussion. A third author (MF) was consulted when agreement could not be reached.

### 2.7. Data Items

For each included source, the following information was charted: reference (authors and year of publication); country; study design; sample size and composition; key findings; methodological limitations; reported post-transplant changes; attribution of changes to donor characteristics; source and nature of donor information; data sources or informants; proposed explanatory frameworks; source category (empirical vs. secondary or non-empirical). For empirical studies, the country referred to the study setting; for secondary and non-empirical sources, it referred to the institutional affiliation of the first author.

### 2.8. Critical Appraisal of Individual Sources of Evidence

The methodological quality of the included sources was assessed using appraisal tools appropriate to each source type. The JBI Critical Appraisal Tools [27] were applied to reviews and evidence syntheses, qualitative studies, case series, cross-sectional studies, and textual evidence, including opinion and conceptual contributions. The Mixed Methods Appraisal Tool (MMAT) [28] was used for mixed-methods studies. Each checklist item was rated using the response options specified by the respective tool. Given the differences in methodological domains and number of items across the JBI and MMAT tools, no overall numerical score or common threshold-based classification was applied across source types; appraisal findings were interpreted at the item and source-type level. Assessments were conducted independently by two reviewers (GA and DN), with disagreements resolved through discussion, and when necessary, consultation with a third reviewer (RL).

Consistent with the scoping review methodology, no source was excluded based on methodological appraisal [23,24,25]. The appraisal findings were used to contextualize interpretation of the evidence, while preserving the review’s objective of mapping the available literature [26].

### 2.9. Synthesis of Results

Consistent with the aims of this scoping review and the framework proposed by Arksey and O’Malley [23], findings were synthesized using a descriptive narrative approach informed by the methodology for narrative synthesis [29,30]. The synthesis was structured according to the three research questions.

First, post-transplant changes reported by HT recipients were mapped across descriptive domains and specific manifestations. Second, each change was classified according to its relationship with donor characteristics as: (a) reported without donor attribution; (b) recipient-perceived donor correspondence without independent verification; or (c) donor–recipient correspondence supported by independently obtained donor information. Third, proposed explanations for these changes and donor–recipient correspondences were mapped across the included sources.

Empirical evidence was distinguished from secondary and non-empirical sources throughout the synthesis. Empirical evidence was considered to address changes and correspondence with donor characteristics. Secondary sources were used only to map proposed explanatory frameworks rather than as confirmation of empirical phenomena. Critical-appraisal findings were considered when interpreting the available evidence. Results are presented narratively and are complemented by tables and visual summaries.

Generative artificial intelligence was used during manuscript preparation and revision solely to support language editing and the refinement of wording and clarity. It was not used for literature searching, study selection, data extraction, critical appraisal, or evidence synthesis. All AI-assisted text was critically reviewed and approved by the authors.

## 3. Results

### 3.1. Selection of Sources of Evidence

**Figure 1:**
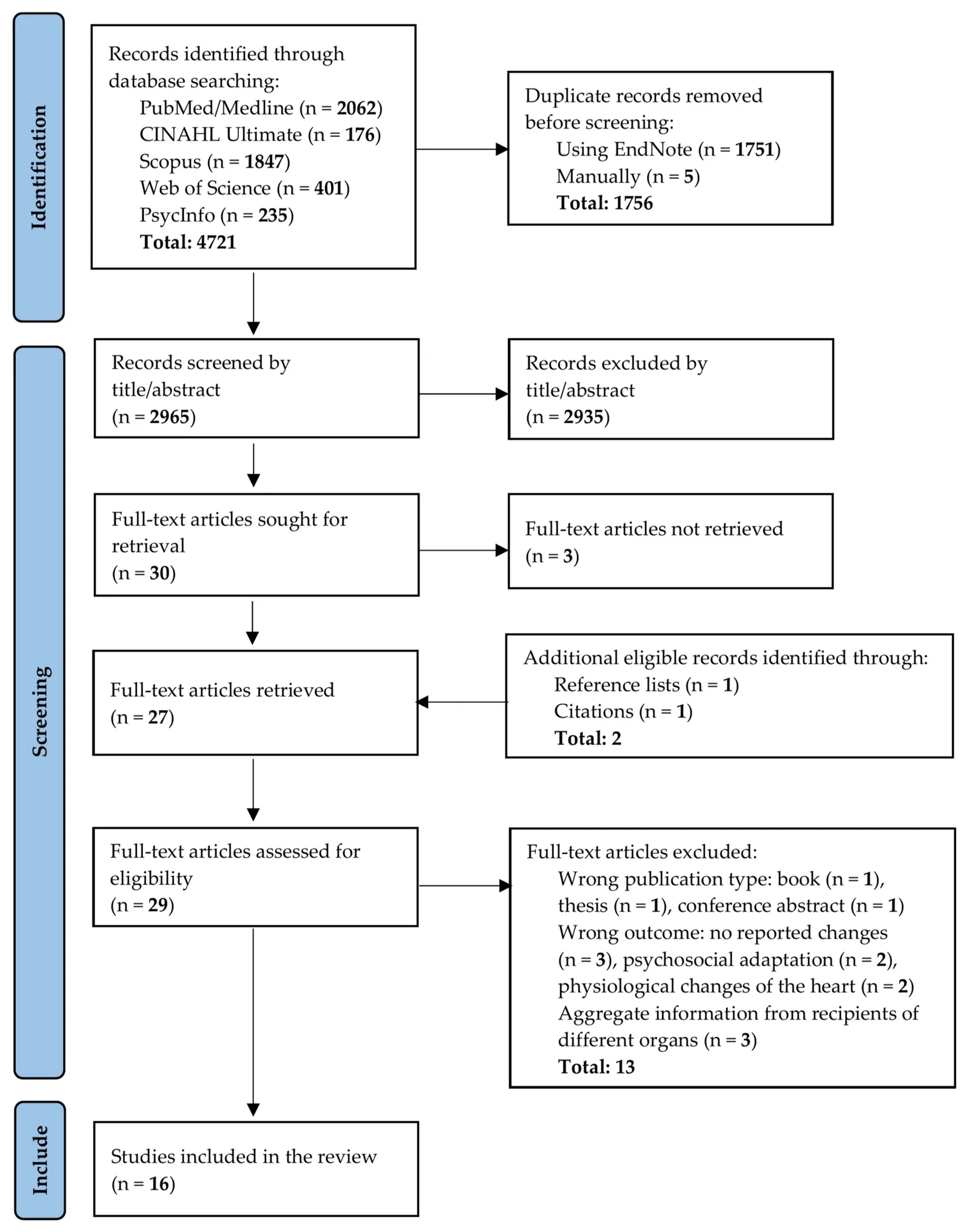
PRISMA flow diagram illustrating the identification, screening, eligibility assessment, and inclusion of sources of evidence.

The database searches identified 4721 records. After the removal of 1756 duplicates, 2965 records underwent title and abstract screening. Of these, 2935 were excluded because they did not meet the eligibility criteria, and 30 reports were sought for full-text retrieval. Three reports could not be retrieved despite support from the institutional library service. Citation searching of the 27 retrieved reports identified two additional potentially eligible reports. Consequently, 29 full-text records were assessed for eligibility, of which 13 were excluded for specific reasons. Ultimately, 16 sources of evidence met the eligibility criteria and were included in the review. The study-selection process is presented in the PRISMA flow diagram [31] (see Figure 1).

### 3.2. Characteristics of Sources of Evidence

The characteristics of the studies included in this review are presented in Table 1.

**Figure 2:**
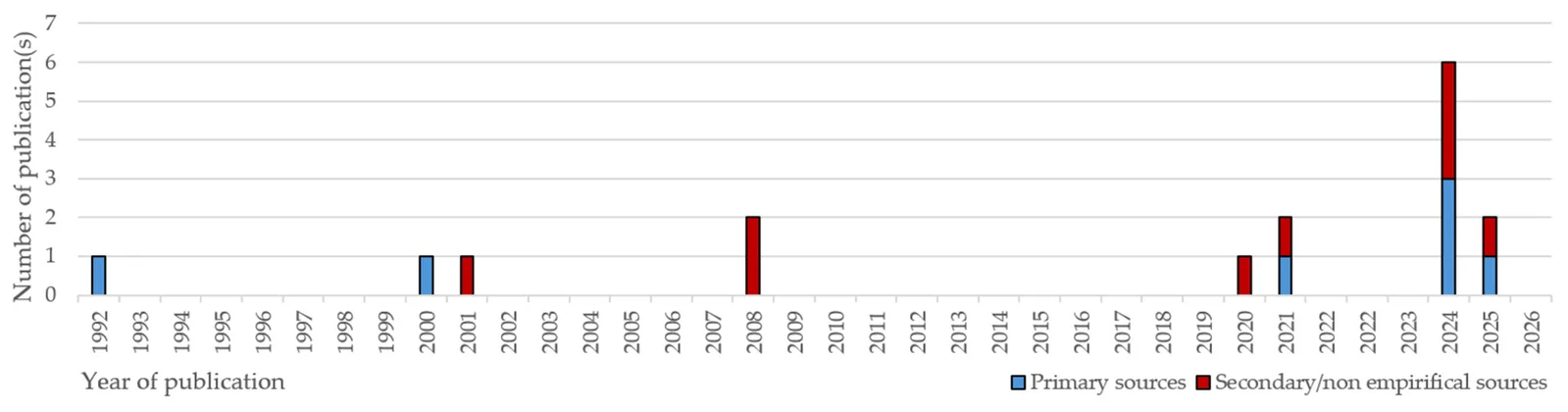
Temporal distribution of included sources by publication year and source type.

The 16 included sources were published between 1992 and 2025, with an uneven distribution over time. Eleven sources (68.7%) were published from 2020 onward [16,18,22,32,33,35,36,37,38,39,40]. The largest number of included sources was published in 2024 (*n* = 6; 37.5%) [18,32,35,36,37,38]. The distribution of the included sources by year of publication is presented in Figure 2.

The included sources showed broad geographical distribution. North America accounted for the largest proportion (*n* = 6; 37.5%), with all sources originating from the United States of America [15,16,35,37,41,42]. Europe contributed three sources (18.8%) from Austria [34], Slovakia [39], and the United Kingdom [43], while three sources (18.8%) originated from the Middle East, namely Iran [40], Saudi Arabia [18], and the United Arab Emirates [32]. One source each (6.3%) originated from East Asia (Taiwan) [38], West Asia (Türkiye) [33], South Asia (India) [36], and Sub-Saharan Africa (Nigeria) [22]. No eligible sources originated from Latin America or Oceania. Within this distribution, Austria [34], India [36], Iran [40], Taiwan [38], Türkiye [33], and two sources from the United States of America [35,41] represent study settings of empirical studies. In contrast, Saudi Arabia [18], the United Arab Emirates [32], Slovakia [39], the United Kingdom [43], Nigeria [22], and four sources from the United States of America [15,16,37,42] represent the first authors’ institutional affiliations of secondary or non-empirical sources.

**Figure 3:**
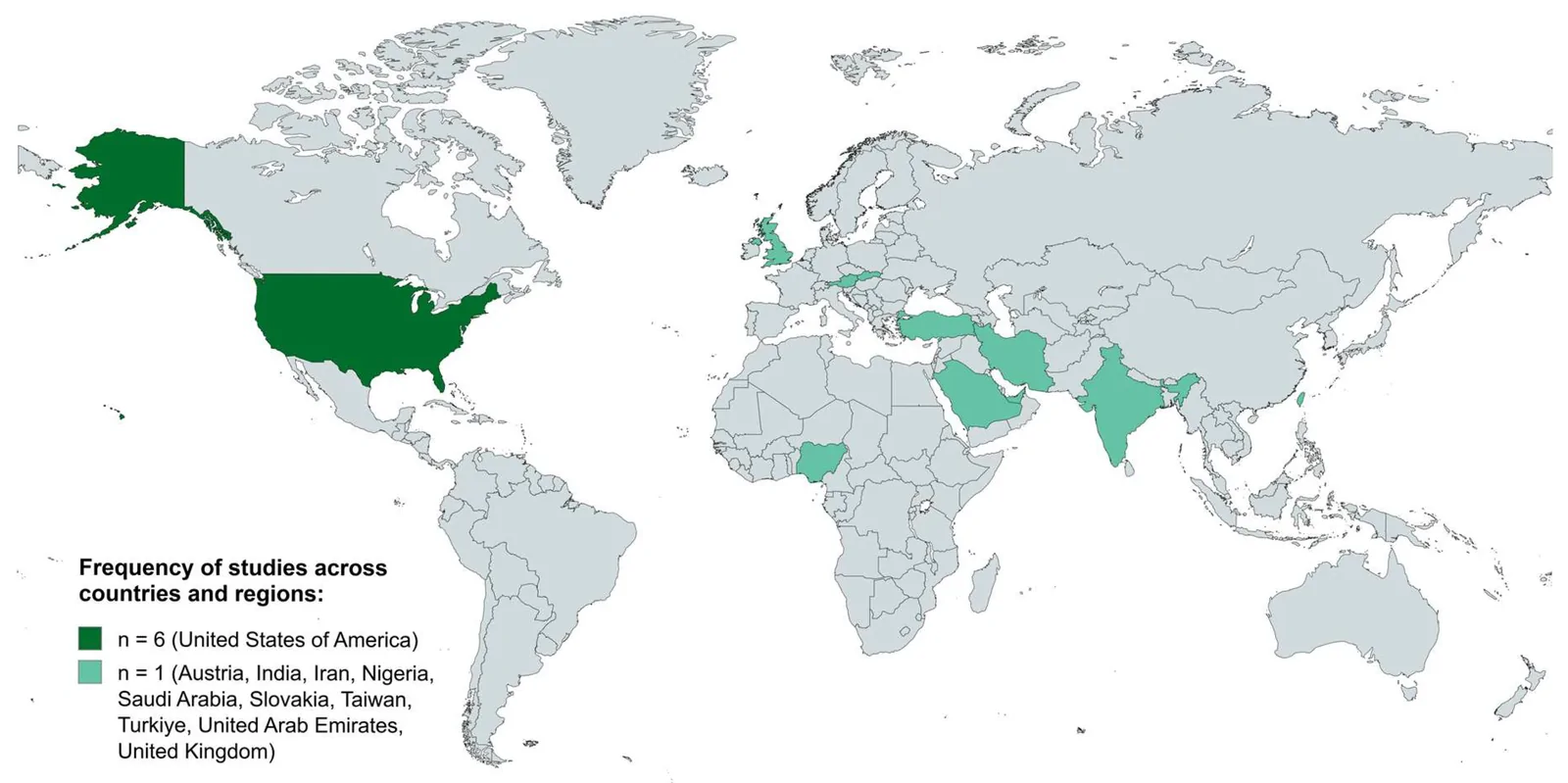
Geographical distribution and frequency of included sources across countries and regions.

The geographical distribution of the included sources is presented in Figure 3.

The included sources were methodologically heterogeneous. Seven (43.8%) were empirical studies [33,34,35,36,38,40,41], two (12.5%) were secondary sources [18,22], and seven (43.8%) were opinions or conceptual contributions [15,16,32,37,39,42,43]. Among the empirical studies, three used qualitative designs [34,36,40], two were cross-sectional studies [33,35], one was a case series [41], and one used a mixed-methods design [38]. The secondary literature comprised one narrative review [18] and one scoping review [22]. The remaining sources included four expert opinions [15,37,42,43] and three conceptual contributions [16,32,39].

Sample characteristics varied in terms of size, population, and the inclusion of informants. Five sources (31.2%) focused exclusively on HT recipients, with sample sizes ranging from 7 to 47 participants [34,36,38,40,41], whereas two (12.5%) also included recipients of other transplanted organs or implanted cardiac devices; their HT subsamples ranged from 23 to 45 participants [33,35]. Among the empirical studies, five (31.2%) included recipients as the sole participant group [33,34,35,36,40], while two (12.5%) also collected data from the recipients’ families and donor relatives [38,41]. Secondary, opinion, and conceptual sources did not recruit participants and relied on previously published literature.

### 3.3. Synthesis of Results

#### 3.3.1. Post-Transplant Changes and Their Relationship with Donor Characteristics

To address the first research question, empirical studies identified a broad range of post-transplant changes across multiple dimensions, including dietary preferences [35,38,40,41], social and occupational functioning [33,36], physical functioning [33,35,36], interests and preferences [34,35,38,40,41], personality and emotions [33,34,35,36,38,40,41], sexuality and gendered behavior [33,35,40,41], identity and embodiment [34,35,36,38,40], memory-, dream-, and sensory-related experiences [35,38,41], and spirituality [34,35,36,40].

Regarding the second research question, the empirical evidence linking these changes to donor characteristics varied. Some changes in personality, temperament, interests, gendered behavior, identity, and spirituality were attributed to the donor by the recipients themselves, but were not independently verified [34,36,40]. In contrast, donor–recipient correspondences supported by donor information were reported for dietary preferences, interests, temperament and behavior, sexuality, and dream-, sensory-, or other unusual experiences [38,41]. These correspondences were examined by comparing recipient accounts with reports from recipient and donor relatives [41], or through matched recipient–donor-family groups and qualitative triangulation [38]. Notably, although some donor–recipient similarities were identified qualitatively, quantitative analysis showed no significant correlations between donor-family assessments and recipient personality ratings in a study [38]. The empirical evidence supporting donor–recipient correspondences was methodologically heterogeneous, with most reports arising from studies with limitations in sampling, retrospective assessment, and the control of alternative explanations.

The reported post-transplant changes and their relationship with the donor characteristics identified across the empirical studies are summarized in Table 2.

#### 3.3.2. Proposed Explanatory Frameworks for Post-Transplant Changes and Donor-Recipient Correspondences

Concerning the third research question, the included literature, encompassing both empirical studies and secondary or non-empirical sources, reflected two broad interpretive levels. Within the empirical studies, post-transplant changes were predominantly interpreted in relation to psychological and existential adaptation, reconstruction of identity and bodily self, medication effects, perioperative and neurocognitive factors, changes in physical capacity, and sociocultural influences on the meaning attributed to the transplanted heart [33,34,35,36,38,40,41]. Primary studies also considered, to varying degrees, donor-related expectations, coincidence, and other interpretive processes when accounting for perceived donor–recipient similarities [34,35,38,41].

Secondary and non-empirical sources further developed these conventional and alternative interpretations, including suggestibility, exposure to donor information, source-memory processes, coincidence, confirmation bias, and retrospective attribution [15,16,22,37,42]. A second group of explanatory frameworks sought more specifically to account for donor–recipient correspondences through hypothetical mechanisms of information retention or transmission. Some of these hypotheses, particularly cellular memory and related intracardiac or energetic explanations, were also discussed or mentioned in primary empirical studies [35,38,40,41]. However, they were developed more extensively in secondary and non-empirical sources, which proposed cellular and molecular memory, extracellular-vesicle-mediated transfer, intracardiac neural processes, energetic or electromagnetic models, quantum-information mechanisms, and nonlocal or transpersonal interpretations [15,16,18,22,32,37,39,42,43]. Overall, the donor-transfer mechanisms identified in the literature remained theoretical or hypothesis-generating.

The detailed explanatory frameworks and the extent to which each was addressed across the empirical, secondary, and non-empirical sources are presented in Table 3.

### 3.4. Methodological Appraisal of Included Sources

The critical appraisal showed considerable heterogeneity across the included literature. Among the empirical studies, recurrent limitations included small or selected samples, retrospective assessment, absence of pre-transplant baseline measures, limited use of validated instruments, susceptibility to recall bias, and insufficient control of alternative explanations [33,34,35,36,38,40,41]. Secondary and non-empirical sources showed different methodological limitations, including non-systematic or insufficiently transparent evidence selection, reliance on anecdotal or secondary material, and theoretical extrapolation beyond directly observed evidence [15,16,18,22,32,37,39,42,43].

Detailed appraisal results are presented in Table 4.

## 4. Discussion

This scoping review provides an integrated account of post-transplant changes reported by adult HT recipients, their attribution to donor characteristics, and the explanatory frameworks proposed to interpret these phenomena. These represent different levels of inference and should not be conflated. Primary empirical studies documented a broad range of changes involving preferences, emotional and personality-related experiences, social and physical functioning, sexuality, identity and embodiment, memory-, dream-, and sensory-related experiences, and spirituality [33,34,35,36,38,40,41]. Some of these changes were interpreted by the recipients as reflecting donor characteristics [34,36,40], whereas a smaller subset involved correspondences supported by independently obtained donor information [38,41]. In contrast, proposed mechanisms of donor–recipient correspondences have been developed predominantly in the secondary, conceptual, and opinion-based literature [15,16,18,22,32,37,39,42,43], although some empirical studies mentioned these hypotheses [35,38,40,41]. This distinction is central to interpreting literature in which phenomenological observations coexist with specific but hypothetical explanatory models.

The reported changes identified in empirical studies are consistent with the broader understanding of HT as a major psychosocial, embodied, and biographical transition. Recipients must adapt to medical uncertainty, fear of rejection, treatment burden, altered social roles, and the persistent vulnerability associated with living with a transplanted organ [5,10,13,44,45]. Emotional, behavioral, and preference-related changes may therefore emerge within a wider process of adaptation following severe illness, major surgery, and the transition from life-threatening disease to long-term survivorship [5,46,47,48]. Psychological distress, including depression, anxiety, and post-traumatic stress symptoms, is documented after HT [6,7,8,9], while fear of graft rejection, uncertainty, medication-related effects, functional changes [49,50,51,52], and the demands of chronic post-transplant care [5,53] may further influence the recipients’ emotional and behavioral adjustment. Within this context, reported changes may reflect adaptation to a profoundly altered life situation rather than phenomena requiring a donor-derived explanation [5,46,48].

Nevertheless, conventional categories of psychological distress alone do not adequately capture the identity- and embodiment-related experiences identified in the empirical literature. Recipients may experience the graft as simultaneously incorporated into the body and biographically associated with another person, thereby challenging established boundaries between the self and other [36,38,40]. Previous research has similarly conceptualized transplantation as a transformative process in which bodily integrity, self-continuity, and personal identity may need to be renegotiated [54,55,56]. Accordingly, changes in self-perception or feelings of unfamiliarity toward the graft should not necessarily be reduced either to psychopathology or to evidence of donor-characteristic transfer; they may represent forms of embodied adaptation and identity reconstruction.

The symbolic and cultural meanings associated with the heart may further influence this process. Across cultural and historical traditions, the heart has been associated with emotion, love, courage, morality, spirituality, and personal essence [57,58]. These meanings coexist with biomedical understandings of the heart and may influence how recipients interpret bodily and psychological post-transplant changes [59]. Recipients may conceptualize the graft as a biological replacement, a gift, or as a material connection with the donor [12,51,60]. Such interpretations are therefore likely to arise from an interaction between embodied experience, individual beliefs, and wider sociocultural frameworks rather than from a single explanatory process.

Existential changes identified in the empirical literature further support this interpretation. Survival following HT may be experienced as rebirth or a second opportunity for life, accompanied by gratitude, renewed appreciation of life, changes in priorities, or greater spiritual engagement [40,47,59,60]. At the same time, survival can coexist with indebtedness, guilt, and concerns about whether one is living in a manner worthy of the donated organ [61,62,63]. For instance, recipients may feel obliged to remain grateful, honor the donor, or preserve an idealized legacy [64], struggling to reconcile their survival with another person’s death [12,65]. The donor therefore occupies a complex position in recipients’ meaning-making, simultaneously associated with loss and survival. These processes may contribute to changes in values, relationships, spirituality, and self-concept without implying the transmission of donor characteristics.

A specific issue concerns the interpretation of perceived donor–recipient correspondences. Although some empirical studies documented recipient-attributed similarities in personality, temperament, interests, gendered behavior, identity, and spirituality [34,36,40], independently sourced donor information was available in only a limited portion of the evidence [38,41]. Such information strengthens the documentation of correspondence but does not establish its cause. This caution is reinforced by the methodological characteristics of the available evidence. Studies providing detailed donor–recipient correspondences were based on small or selected samples, retrospective accounts, and limited standardized assessment, with potential susceptibility to recall, matching, and confirmation biases [38,41]. Collateral accounts from recipients and donor relatives do not resolve these limitations. Independent informants may strengthen confidence that a reported characteristic was not generated solely by the recipient [38,41], yet agreement across informants remains vulnerable to retrospective interpretation and selective matching [15,22,37,41]. Moreover, donor relatives may seek continuity with the deceased donor, while recipients may reinterpret their own experiences after learning donor characteristics [15,22,37]. Previous research suggests that donor families can experience an enduring symbolic connection with recipients and may perceive donor characteristics in them [66]. Contact between recipients and donor families can therefore be meaningful while also shaping subsequent interpretations of the graft and donor–recipient relationship [66,67,68].

The current evidence therefore supports the existence of reported and perceived donor–recipient correspondences, but not the conclusion that such similarities reflect the transmission of donor-derived information.

The explanatory literature further illustrates why empirical observation and causal interpretation must remain distinct. Several frameworks account for post-transplant changes without requiring donor-derived transfer, including psychological and existential adaptation, graft incorporation, medication effects, perioperative factors, sociocultural influences, suggestibility, exposure to donor information, coincidence, and attributional processes [15,22,34,35,36,37,38,40,41,42]. These processes are not mutually exclusive and may interact. An experience may, for example, initially be perceived as unexpected, later acquire significance after exposure to donor information, and subsequently be incorporated into the recipient’s narrative of transplantation. Such a sequence does not invalidate the experience but demonstrates why the experience itself should be distinguished from its causal attribution.

Other frameworks propose that donor-related information could be retained or transmitted through cellular, molecular, neurocardiac, energetic, quantum, or nonlocal processes [15,16,18,22,32,35,37,39,41,42,43]. Some invoke biological phenomena, including epigenetic regulation, extracellular-vesicle signaling, RNA-mediated communication, and bidirectional heart–brain signaling [16,18,22,35,37,39]. However, the existence of these biological processes does not demonstrate that autobiographical memories, preferences, personality traits, or other donor-specific psychological information can be encoded in the heart and subsequently expressed by the recipient. Epigenetic memory, for example, concerns the persistence of regulatory cellular states and should not be equated with autobiographical or declarative memory [17]. Similarly, extracellular vesicles and RNA can mediate intercellular communication, but their proposed role in transmitting donor memory after HT remains hypothetical [16,18,22,39].

The structure and methodological appraisal of the literature reinforce this distinction. Although donor-transfer hypotheses were occasionally mentioned or discussed in primary empirical studies [35,38,40,41], their detailed development occurred in secondary and non-empirical sources [15,16,18,22,32,37,39,42,43]. Several of these contributions rely on anecdotal observations, theoretical extrapolation, or selective secondary evidence. Repetition of the same mechanism across conceptual or secondary publications should therefore not be interpreted as empirical corroboration. These frameworks are more appropriately regarded as hypotheses mapped within the literature than as established explanations for the reported phenomena.

A distinction can therefore be made between the phenomenological reality of post-transplant experiences and the ontological claims proposed to explain them. Recipients may genuinely experience unfamiliar preferences, emotions, dreams, bodily sensations, changes in identity, or a perceived connection with the donor. Such experiences may have substantial psychological, relational, and clinical significance regardless of whether their attributed cause is supported. Conversely, an ontological claim that donor memories, personality, or identity persist within the transplanted heart and are transmitted to the recipient requires a stronger level of evidence than is currently available. Maintaining this distinction avoids two opposing errors: interpreting unusual post-transplant experiences as proof of donor-memory transfer, and dismissing recipients’ accounts because the proposed explanation remains unsupported. A scientifically cautious approach can acknowledge the experience while remaining neutral regarding causal interpretations that have not been demonstrated.

### 4.1. Implications for Practice and Research

The findings support post-HT follow-up that addresses physical and psychological outcomes together with identity, embodiment, existential concerns, and donor-related meaning. Healthcare professionals should be aware of the challenges experienced by HT recipients [47,51] and should be prepared to explore unexpected emotional, behavioral, and sensory experiences, including guilt and donor-related concerns [63]. These experiences should be acknowledged without pathologizing recipients or endorsing unsupported explanations. To facilitate person-centered care, Table A2 provides suggested questions addressing emotional adjustment, identity and embodiment, donor-related meaning, unusual experiences, and their potential impact on functioning and treatment. These prompts are intended to support clinical conversation rather than constitute a validated screening instrument. Nurses are well-positioned to identify distress, support meaning-making, and coordinate referral to psychological, social, or spiritual care when appropriate. Advanced practice nursing roles may further strengthen continuity, assessment, and multidisciplinary management [69,70,71], consistent with international recommendations for longitudinal and multidisciplinary care after HT [72,73].

Future research should clearly distinguish the reported experience, its attribution to donor characteristics, and any proposed explanatory framework. Prospective, multicenter, and longitudinal designs should document relevant recipient characteristics and experiences before and after transplantation, preferably before the disclosure of donor information, and compare subsequent reports with independently obtained donor data using predefined criteria. Studies should also assess and report recipients’ exposure to donor information. Mixed-methods approaches may help clarify how these experiences develop and relate to psychological well-being, identity integration, adherence, relationships, and quality of life. Nursing theories may provide useful conceptual frameworks for interpreting recipients’ adaptation and lived experiences [3]. Greater cultural diversity is also needed to examine how social, spiritual, and symbolic meanings shape post-transplant interpretation and adaptation.

### 4.2. Limitations

Several limitations should be considered. First, the evidence base was methodologically heterogeneous. Only seven included sources were primary empirical studies; the remaining were secondary or non-empirical contributions. Although their inclusion was appropriate to the scoping objective of mapping both reported phenomena and proposed explanations, these source types provide different levels of evidence. Accordingly, secondary and non-empirical sources were used to contextualize explanatory frameworks rather than as independent confirmation of empirical findings. Second, most empirical studies relied on small samples, with five of the seven primary empirical studies including fewer than 30 HT recipients, alongside retrospective self-report and the absence of pre-transplant baseline assessments. These limitations are relevant to perceived donor–recipient correspondences, which may be susceptible to recall, selection, matching, confirmation, and attribution biases. Independently obtained donor information was available in only two studies, and the timing and extent of the recipients’ prior exposure to donor characteristics were not always clearly established. Therefore, the available evidence cannot determine whether reported similarities exceed chance or establish a causal mechanism of donor-derived information transfer. Third, methodological appraisal identifies important limitations across the included literature, including sampling and measurement limitations in empirical studies and limited methodological transparency, selective evidence use, or theoretical extrapolation in several secondary and non-empirical sources. Fourth, publication and reporting bias cannot be excluded, as unusual experiences may be preferentially reported. Potential overlap between primary cases and their subsequent discussion in other sources should also be considered. Given the heterogeneous designs and non-probability samples, the prevalence of these experiences cannot be estimated from the available evidence. Finally, the geographical distribution of the evidence was uneven, with no eligible sources identified from Latin America or Oceania and several regions represented by only one study. This may limit the transferability of the findings given the potential influence of cultural, spiritual, and symbolic meanings of the heart. Although the search covered five major databases, imposed no language or date restrictions, and was supplemented by backward and forward citation searching, three potentially eligible reports could not be retrieved. These limitations should be considered when interpreting the findings.

## 5. Conclusions

This scoping review identified a broad range of behavioral, emotional, identity-related, sensory, and existential changes reported after HT. Some recipients perceived these changes as corresponding to donor characteristics; however, the available evidence was limited by retrospective reporting, subjective attribution, and methodological heterogeneity. The available evidence does not establish the transfer of donor memories, personality, or identity. These experiences should therefore be understood primarily as clinically and psychologically meaningful aspects of post-transplant adaptation rather than as evidence of donor-derived information transfer. Their recognition may support more person-centered, psychologically informed, and multidisciplinary follow-up. Future prospective, longitudinal, and culturally diverse research using predefined assessments and independently obtained donor information is needed to clarify the occurrence, interpretation, and clinical significance of these experiences.

## Figures and Tables

**Table 1:** Characteristics of the included sources of evidence.

Reference	Country	Study Design	Sample	Key Findings	Limitations
[18]	Saudi Arabia	Narrative review	–	Reports changes in preferences, emotions, personality, identity, and memory; discusses cellular/epigenetic memory, intracardiac neural networks, exosomes, and energetic mechanisms.	Non-systematic evidence selection; no reproducible search/selection process or formal critical appraisal.
[32]	United Arab Emirates	Conceptual contribution	–	Proposes molecular polarization and spin as mechanisms for storing and transferring information that could influence personality and memory after transplantation.	No empirical testing; model based on theoretical assumptions and extrapolation; no direct evidence linking the proposed mechanisms to memory or personality.
[22]	Nigeria	Scoping review	–	Synthesizes neurocognitive, personality, and memory changes; concludes that donor-derived memory transfer remains inconclusive and likely multifactorial.	Limited methodological detail and non-standardized quality assessment; heterogeneous evidence base.
[33]	Türkiye	Cross-sectional study	66 adult recipients (45 HT and 21 LVAD)	HT recipients reported depression (34.1%), anger (29.5%), personality/behavioral changes (27.3%), and anxiety (22.7%). Social, occupational, lifestyle, and sexual changes were reported; 66.7% reported lifestyle changes, and 60% had stopped working.	Single-center, small comparative sample; non-validated assessment of psychosocial changes; no donor-characteristic assessment.
[34]	Austria	Qualitative study	47 adult HT recipients	79% reported no personality change; 15% reported non-donor-attributed changes in priorities, preferences, and outlook; 6% attributed personality changes to the new heart.	Single-center, small, male-predominant sample; retrospective self-report; no validated personality measure or pre-transplant baseline; potentially suggestive interview; no donor corroboration.
[35]	United States of America	Cross-sectional study	47 adult recipients (23 HT and 24 other organ)	Most HT recipients (91.3%) reported personality-related changes involving temperament, emotions, food, activities, identity, spirituality, sexuality, and memory.	Small convenience sample; selection bias from recruitment strategy; retrospective self-report; no validated personality measure or pre-transplant baseline; no donor or collateral corroboration.
[36]	India	Qualitative study	23 adult HT recipients	Some recipients perceived acquisition of donor personality traits, experienced the heart as initially foreign, imagined donor characteristics, and reported major lifestyle, relational, occupational, and spiritual changes.	Single-center, small sample; telephone interviews limiting non-verbal observation; self-reported donor-related perceptions; no independent donor corroboration.
[15]	United States of America	Opinion	–	Discusses donor-related changes and contrasts conventional and cellular-memory explanations with a nonlocal-consciousness interpretation.	Selective evidence base; reliance on anecdotal and secondary reports; no empirical testing.
[37]	United States of America	Opinion	–	Discusses donor-like changes and considers medication, psychological adaptation, restored health, and coincidence alongside cellular/non-synaptic memory hypotheses.	Selective evidence base; reliance on anecdotal and secondary reports; no empirical testing.
[38]	Taiwan	Mixed-methods study	20 adult HT recipients	Identifies qualitative donor–recipient similarities in diet, emotions/temperament, dreams, and unusual experiences; 38% had ≥2 corresponding aspects. 15% experienced the new heart as not their own. BFI-19 correlations were non-significant	Small matched sample; investigator-judged correspondence; donor traits reported indirectly by relatives; retrospective recall; limited quantitative assessment.
[39]	Slovakia	Conceptual contribution	–	Proposes exosome-mediated transfer of donor memory engrams, including RNA and other molecular cargo, from donor brain to heart and subsequently to the recipient brain.	No direct empirical testing in HT recipients; mechanism extrapolated from observational and experimental evidence; hypothesis-generating only.
[16]	United States of America	Conceptual contribution	–	Categorizes changes in preferences, temperament, identity, and donor-life memories; proposes cellular, intracardiac neurological, and energetic memory mechanisms.	Non-systematic, selective evidence base; reliance on anecdotal/case reports; proposed mechanisms not empirically tested in HT recipients.
[40]	Iran	Qualitative study	13 adult HT recipients	Reports changes in temperament, food/color preferences, religiosity, gender-related characteristics, identity, and perceived donor–recipient unity; some changes were subjectively linked to the donor.	Single-center, small purposive sample; retrospective self-report; potential recall and social-desirability bias; no independent donor corroboration.
[41]	United States of America	Case series	7 adult HT recipients	Reports 2–5 donor–recipient correspondences per case across preferences, behaviors, fears, names, and sensory/dream experiences; cellular/systemic memory is proposed as a possible explanation.	Small selected case series; no standardized assessment; potential selection, recall, matching, and confirmation bias despite collateral reports.
[42]	United States of America	Opinion	–	Reviews donor-associated changes and case observations; considers conventional alternatives while arguing that cellular/systemic memory warrants further investigation.	Anecdotal, non-systematic evidence; no empirical testing of the proposed mechanisms.
[43]	United Kingdom	Opinion	–	Reviews donor-like preference and behavioral changes and discusses cellular/systemic memory, heart consciousness, and electrophysiological resonance.	Brief opinion; selective secondary evidence; reliance on anecdotes; no empirical testing.

Legend: HT = heart transplantation; LVAD = left ventricular assist device.

**Table 2:** Reported post-transplant changes and their relationship with donor characteristics.

Domain	Reported Post-Transplant Changes	Donor Correspondence
Food and dietary preferences	Change in food preferences, not further specified [35]; new desire for lettuce and cabbage despite previously not eating them [40]	*
	New preference for spicy food, rejection of certain foods [38]; new meat aversion/vegetarianism and nausea/vomiting triggered by meat [41]	***
Social and occupational functioning	Impaired communication with friends and colleagues, impaired family relationships, social isolation [33]; relationship deterioration [36]	*
	Changing profession [36]; stopping work, early retirement, job loss, reduced productivity, difficulty in finding employment [33]	*
Physical functioning	Changes in physical activity, not further specified [35]; reduced physical activity [33]; difficulties with exercise, running, and household activities [36]	*
Interests and preferences	Changes in participation in or watching sports and in movies/TV preferences, not further specified [35]	*
	New preference for loud music and headphones; new interest in stereos and cars [34]	**
	New love of classical music; new interest in playing guitar; new interest in museums and paintings; new interest in dance and singing [41]	***
	Favorite color changing from red to coral/turquoise [40]	*
	Becoming fonder of gray and white [38]	***
	Changed preferences for people, events, or objects [38]; new interest in nursing/medicine and corresponding changes in educational plans [41]	***
Personality and emotions	Changes in personality and behavior, not further specified [33,35]	*
	Perceived acquisition of donor personality traits and beginning to “behave like the donor” [36]; altered sleep pattern interpreted as reflecting a “night person” donor [34]	**
	Jumping around, new high-pitched giggle, and an adolescent or teenage-like behavioral style [41]	***
	Changes in temperament and emotions, not further specified [35]; depression; anger; anxiety; fear/fear of death, and uncertainty about the future [33]	*
	Becoming calmer [34,40]	**
	Becoming more gentle, thoughtful, “civilized”, with improved temper and greater attentiveness to others [38]	***
Sexuality and gendered behavior	Changes in sexual preferences, not further specified [35]	*
	Shift in sexual attraction toward men [41]	***
	Decreased sexual desire; reduced frequency of intercourse; erectile dysfunction; lack of pleasure during sexual activity; menstrual irregularities [33]	*
	Feeling stronger and courageous after receiving a male heart; reporting greater understanding of boys and behaving more like them [40]	**
	Increased sensuality and perceived knowledge of the female body; new behaviors perceived as feminine (e.g., shopping, purse-carrying) [41]	***
Identity and embodiment	Changes in personal identity, not further specified [35]	*
	Feeling that “someone else’s heart” was beating inside the body [36]; perception that the transplanted heart was not one’s own organ [38]	*
	Perceived physical and emotional unity with the donor [40]; sense that the donor remained present within the recipient and experience of “living two lives” [34]	**
Memory, dreams, and sensory experiences	Changes in memories, not further specified [35]	*
	Apparent ability to complete phrases of songs associated with the donor after hearing the donor’s music [41]	***
	Recurrent dreams/daydreams involving a flash of light and facial heat corresponding to the circumstances of the donor’s fatal injury [41]; dreams involving being chased, falling from a mountain, or being killed by a car, reported as corresponding with donor-related events [38]	***
	Recurrent bodily sensation resembling the impact experienced in the donor’s fatal accident [41]; hearing a young girl’s voice; persistent sensation that something had dropped; increased sensitivity to supernatural events, reported by investigators as corresponding with donor information [38]	***
Spirituality and meaning	Changes in spirituality, not further specified [35]; increased faith in God and religious practice; gratitude for a second life/rebirth [36]	*
	Increased religiosity and religious observance attributed by the recipient to the donor’s strong religious faith [40]	**

Legend: * = reported post-transplant change without donor attribution; ** = recipient-perceived donor correspondence without independent corroboration; *** = donor–recipient correspondence supported by independently obtained donor information.

**Table 3:** Proposed explanatory frameworks for post-transplant changes and donor-recipient correspondences.

Explanatory Framework	Proposed Mechanism or Interpretation	References
Psychological, psychosocial, and identity-related explanations		
Psychological and existential adaptation	Severe illness, transplantation, confrontation with mortality, and recovery may change priorities, emotions, behavior, self-perception, and lifestyle.	[22,34,36,37,38,40] ** [33,42] *
Graft incorporation and identity integration	Incorporation of a foreign organ may disrupt self–other boundaries and require reconstruction of body image, identity, and organ ownership.	[34,36,38,40] **
Donor incorporation fantasies and magical thinking	Recipients may imagine that the donor’s characteristics are incorporated with the organ, producing perceived changes in feelings, personality, or identity.	[22,34,35] ** [16] *
Suggestibility, placebo effects, and psychological association	Expectations, beliefs, and emotionally salient associations with the donor may shape subsequent behavior or the interpretation of perceived changes.	[15,22] ** [38] *
Donor-information exposure and source-memory processes	Information about the donor may be acquired consciously or inadvertently and subsequently influence the recipients’ interpretations of post-transplant experiences; source-memory errors or subconscious processing are proposed to create an impression of donor-derived knowledge.	[15,22,37] ** [16,42] *
Coincidence, confirmation, and attribution biases	Apparent donor–recipient similarities may arise by chance, while confirmation bias, selective recall, and retrospective attribution may increase the salience of matching characteristics relative to non-matching characteristics.	[15,22,37,41] ** [42] *
Pre-existing psychopathology or forgotten personal trauma	Unusual post-transplant experiences may originate from pre-existing psychological vulnerability or re-emergence of the recipient’s own previous experiences.	[16,41,42] *
Sociocultural and symbolic meaning of the heart	Cultural, spiritual, and social beliefs concerning the heart, the donor, and transplantation may shape how recipients interpret bodily, psychological, and identity-related changes.	[15,34,36,38,40,43] **
Deliberate behavioral and lifestyle adjustment	Some apparent post-transplant changes may reflect conscious adaptations to health needs or social circumstances.	[38] **
Pharmacological, physiological, and neurocognitive explanations		
Immunosuppressant and other medication effects	Immunosuppressants and other post-transplant drugs may alter mood, cognition, perception, behavior, appetite, or sleep, thereby mimicking personality or memory changes.	[15,22,34,37,38] ** [16,33,35,36,41,42] *
Immunosuppression-facilitated access to cellular memory	Immunosuppressive treatment is hypothesized to lower the recipient’s threshold for registering information supposedly retained in transplanted tissues.	[41] **
Medication- and diet-related taste changes	Tacrolimus, corticosteroids, altered salivation or digestion, and prolonged hospital diets may change taste and food preferences subsequently attributed to the donor.	[15,37,38] **
Perioperative stress, anesthesia, sleep disruption, and delirium	Surgery, acute stress, anesthesia, disrupted sleep, and postoperative delirium may produce transient cognitive changes, vivid dreams, or unusual perceptions.	[22,38] ** [42] *
Restored hemodynamics and physical capacity	Improved cardiac output, energy, exercise tolerance, and cerebral perfusion may enable new activities or behaviors, creating an impression of personality change.	[22,35,37] **
Vascular, metabolic, inflammatory, and immune–brain mechanisms	Cerebral hypoperfusion, vascular and metabolic comorbidities, systemic inflammation, neuroinflammation, and cytokine-mediated immune–brain signaling may alter cognition, mood, and behavior.	[22] **
Cellular and molecular memory hypotheses		
Cellular memory	Donor-related information or experiences are hypothesized to be encoded outside the brain in cells of the transplanted organ and subsequently expressed in the recipient.	[15,16,18,22,35,37,41,42,43] ** [38,40] *
Epigenetic memory	Persistent DNA methylation, histone modification, and related epigenetic changes may encode environmentally acquired information in donor cells and persist after transplantation.	[16,18,22,37] ** [35,38] *
DNA/genomic memory	DNA is proposed as an information-storage substrate; donor-derived genomic material could theoretically convey information to recipient cells	[16,18,22,37] ** [35] *
RNA/non-coding RNA memory	Coding and non-coding RNAs may encode or transmit memory-related information and modify cellular function in the recipient.	[16,18,22,37,39] ** [35,38] *
Protein and prion-like memory	Stable proteins or prions may participate in persistent information storage and potentially intercellular transmission.	[16,18,22] ** [35,39] *
Exosome/extracellular-vesicle-mediated transfer	Extracellular vesicles may transport RNA, DNA, proteins, and signaling molecules from donor cardiac cells through the circulation into the recipient brain.	[16,18,22,35,37,39] **
Engram transfer via exosomes	Memory engrams are hypothesized to form in the donor brain, reach or persist in the heart, and be retransmitted from the transplanted heart to the recipient brain via exosomes.	[22,35,39] **
Neurocardiac and bio-informational hypotheses		
Intracardiac neurological memory/“heart brain”	The intrinsic cardiac nervous system may encode and retain information; because intracardiac neurons are transplanted with the heart, information could theoretically accompany the organ.	[15,16,18,22,35] ** [38] *
Heart–brain neural communication	Afferent and efferent neural pathways permit cardiac signals to influence brain regions involved in emotion and behavior.	[15,18,22,39] **
Neurohumoral and neuropeptide signaling	Cardiac neurotransmitters, neuropeptides, hormones, and autonomic signaling may influence stress regulation, mood, bonding, cognition, and behavior.	[18,22] **
Intracardiac neuroplasticity and neural remodeling	Post-transplant denervation, partial reinnervation, and remodeling of cardiac neural networks may modify heart–brain signaling and potentially neuropsychological processing.	[16,22] **
Systemic, energetic, and quantum-information hypotheses		
Systemic memory/living-systems theory	Dynamic systems containing recurrent feedback loops are proposed to store information and energy; transplanted tissue could therefore retain aspects of the donor’s systemic history.	[41,42,43] ** [15] *
Energetic/electromagnetic memory	Donor-related information is hypothesized to be encoded in the energetic or electromagnetic properties of the heart and transferred with the organ.	[16,18,22,35,41,43] ** [38] *
Electromagnetic resonance/electrophysiological mirroring	Electromagnetic or electrophysiological coupling is proposed to permit information exchange or synchronization between cardiac and neural activity, or between individuals.	[41,43] **
Microtubule-based memory	Microtubules are proposed as subcellular structures capable of storing or processing information beyond conventional synaptic mechanisms.	[41,42] * [32] **
Orch OR/quantum polarization–spin model	Information is proposed to be encoded as molecular polarization and electron/photon spin states in microtubules and analogous structures in heart cells, proteins, DNA, and RNA; brain-derived information may be copied to the heart through blood cells, neural pathways, and electromagnetic waves and subsequently transferred with the organ.	[32] **
Spiritual, non-local, and transpersonal explanations		
Spirituality, rebirth, self-transcendence, and meaning making	Transplantation may be experienced as a second life or existential gift, increasing faith, gratitude, optimism, self-transcendence, and meaning, thereby changing attitudes and behavior.	[36,38,40] **
Intensified consciousness following near-death/transplantation	Near-death trauma and the extraordinary significance of receiving a new life may intensify a normally latent consciousness connection with the donor.	[15,42] **
Nonlocal mind/donor–recipient nonlocal consciousness	Consciousness is proposed not to be confined to the brain, body, space, or time; donor–recipient information exchange could therefore occur without physical storage of memory in the transplanted tissue.	[15] ** [16] *
Telesomatic phenomena/empathic resonance	Thoughts, sensations, or bodily experiences may be shared between individuals outside conventional sensory pathways, potentially accounting for donor–recipient parallels.	[15] **
Reincarnation/persistence of consciousness after death	Memory, personality, or consciousness may persist independently of the physical body; transplant phenomena are interpreted as potentially related to this broader persistence.	[15] **
Heart consciousness/dual consciousness	The heart is conceptualized as possessing consciousness-like properties, raising the possibility that transplantation introduces aspects of another consciousness together with the organ.	[43] **

Legend: ** = sources that propose, develop, or discuss the explanatory framework; * = sources that cite or mention the explanatory framework without substantive discussion.

**Table 4:** Methodological appraisal of included sources of evidence.

Reference	Q1	Q2	Q3	Q4	Q5	Q6	Q7	Q8	Q9	Q10	Q11
Reviews (assessed using the JBI checklist for systematic reviews and research syntheses, 11 items)											
[18]	U	U	U	U	U	U	U	U	U	Y	Y
[22]	U	Y	U	Y	U	U	U	U	U	Y	Y
Qualitative studies (assessed using the JBI checklist for qualitative research, 10 items)											
[34]	U	Y	Y	Y	Y	U	U	Y	N	Y	-
[36]	U	Y	Y	Y	Y	U	U	Y	Y	Y	-
[40]	U	Y	Y	Y	Y	U	U	Y	Y	Y	-
Case series (assessed using the JBI checklist for case series, 10 items)											
[41]	Y	Y	U	U	U	Y	Y	U	U	U	-
Cross-sectional studies (assessed using the JBI checklist for analytical cross-sectional study, 8 items)											
[33]	Y	Y	U	Y	U	Y	Y	Y	-	-	-
[35]	Y	Y	U	Y	U	U	Y	Y	-	-	-
Opinions/Conceptual contributions (assessed using the JBI checklist for textual evidence, 6 items)											
[32]	Y	U	U	Y	Y	U	-	-	-	-	-
[15]	Y	Y	Y	Y	Y	Y	-	-	-	-	-
[37]	Y	U	U	Y	Y	Y	-	-	-	-	-
[39]	Y	U	Y	Y	Y	Y	-	-	-	-	-
[16]	Y	U	Y	Y	Y	Y	-	-	-	-	-
[42]	Y	Y	Y	Y	Y	Y	-	-	-	-	-
[43]	Y	Y	Y	U	Y	U	-	-	-	-	-
Mixed-methods study (assessed using the Mixed Methods Appraisal Tool, 5 items)											
[38]	Y	Y	Y	U	Y	-	-	-	-	-	-

Legend: JBI = Joanna Briggs Institute; Y = Yes; N = No; U = Unclear.

## Data Availability

No new primary data were generated. Data supporting the findings of this review were extracted from the published sources cited in the article. The completed data-charting form is available from the corresponding author upon reasonable request.

## Supplementary Materials

The following supporting information can be downloaded at https://www.mdpi.com/article/10.3390/jcm15176584/s1, PRISMA-ScR checklist [26].

## Abbreviations

The following abbreviations are used in this manuscript:

HT	Heart Transplantation
JBI	Joanna Briggs Institute
LVAD	Left Ventricular Assist Device
MMAT	Mixed Methods Appraisal Tool

## Appendix A

**Table A1:** Full search strategies for each database.

ID	Search Strategy for PubMed (Search Conducted on 10 August 2026)	Results
1	(“Heart Transplantation”[Mesh] OR heart transplant*[tiab] OR cardiac transplant*[tiab] OR heart recipient*[tiab] OR cardiac recipient*[tiab])	56,545
2	(“Personality”[Mesh] OR “Self Concept”[Mesh] OR “Adaptation, Psychological”[Mesh] OR “Emotions”[Mesh] OR “Temperament”[Mesh] OR “Memory”[Mesh] OR “Body Image”[Mesh] OR “Food Preferences”[Mesh] OR personali*[tiab] OR identit*[tiab] OR “self concept”[tiab] OR “self-concept”[tiab] OR “self identity”[tiab] OR “self-identity”[tiab] OR “sense of self”[tiab] OR selfhood[tiab] OR perception*[tiab] OR behavio*[tiab] OR emotion*[tiab] OR temperament*[tiab] OR preference*[tiab] OR “food preference*”[tiab] OR taste*[tiab] OR craving*[tiab] OR habit*[tiab] OR memor*[tiab] OR “cellular memory”[tiab] OR “memory transfer”[tiab] OR “memory-behavioral transfer”[tiab] OR “memory behavioural transfer”[tiab] OR “personality transfer”[tiab] OR “behavioral transfer”[tiab] OR “behavioural transfer”[tiab] OR “donor personality”[tiab] OR “donor characteristic*”[tiab] OR “donor trait*”[tiab] OR “personality change*”[tiab] OR “identity change*”[tiab] OR “behavioral change*”[tiab] OR “behavioural change*”[tiab] OR “emotional change*”[tiab] OR “psychological change*”[tiab] OR “psychosocial change*”[tiab] OR “psychological adaptation”[tiab] OR “psychological adjustment”[tiab] OR “psychological impact”[tiab] OR “identity dissonance”[tiab] OR “neurocognitive change*”[tiab] OR “neurocognitive outcome*”[tiab] OR embodiment[tiab] OR embodied[tiab] OR “bodily integrity”[tiab] OR “graft incorporation”[tiab])	4,260,780
3	((“Heart Transplantation”[Mesh] OR heart transplant*[tiab] OR cardiac transplant*[tiab] OR heart recipient*[tiab] OR cardiac recipient*[tiab])) AND ((“Personality”[Mesh] OR “Self Concept”[Mesh] OR “Adaptation, Psychological”[Mesh] OR “Emotions”[Mesh] OR “Temperament”[Mesh] OR “Memory”[Mesh] OR “Body Image”[Mesh] OR “Food Preferences”[Mesh] OR personali*[tiab] OR identit*[tiab] OR “self concept”[tiab] OR “self-concept”[tiab] OR “self identity”[tiab] OR “self-identity”[tiab] OR “sense of self”[tiab] OR selfhood[tiab] OR perception*[tiab] OR behavio*[tiab] OR emotion*[tiab] OR temperament*[tiab] OR preference*[tiab] OR “food preference*”[tiab] OR taste*[tiab] OR craving*[tiab] OR habit*[tiab] OR memor*[tiab] OR “cellular memory”[tiab] OR “memory transfer”[tiab] OR “memory-behavioral transfer”[tiab] OR “memory behavioural transfer”[tiab] OR “personality transfer”[tiab] OR “behavioral transfer”[tiab] OR “behavioural transfer”[tiab] OR “donor personality”[tiab] OR “donor characteristic*”[tiab] OR “donor trait*”[tiab] OR “personality change*”[tiab] OR “identity change*”[tiab] OR “behavioral change*”[tiab] OR “behavioural change*”[tiab] OR “emotional change*”[tiab] OR “psychological change*”[tiab] OR “psychosocial change*”[tiab] OR “psychological adaptation”[tiab] OR “psychological adjustment”[tiab] OR “psychological impact”[tiab] OR “identity dissonance”[tiab] OR “neurocognitive change*”[tiab] OR “neurocognitive outcome*”[tiab] OR embodiment[tiab] OR embodied[tiab] OR “bodily integrity”[tiab] OR “graft incorporation”[tiab]))	2062
**ID**	**Search Strategy for CINAHL Ultimate via EBSCOhost (Search Conducted on 10 August 2026)**	**Results**
1	MH “Heart Transplantation” OR TI (“heart transplant*” OR “cardiac transplant*” OR “heart transplant recipient*” OR “cardiac transplant recipient*” OR “heart recipient*” OR “cardiac recipient*”) OR AB (“heart transplant*” OR “cardiac transplant*” OR “heart transplant recipient*” OR “cardiac transplant recipient*” OR “heart recipient*” OR “cardiac recipient*”)	8979
2	MH “Personality” OR MH “Self Concept” OR MH “Adaptation, Psychological” OR MH “Emotions” OR MH “Temperament” OR MH “Memory” OR MH “Body Image” OR MH “Life Change Events” OR MH “Food Preferences” OR TI (personali* OR identit* OR “self concept” OR “self-concept” OR “self identity” OR “self-identity” OR “sense of self” OR selfhood OR “body image” OR embodiment OR embodied OR existential* OR “personality change*” OR “identity change*” OR “behavioral change*” OR “behavioural change*” OR “emotional change*” OR “psychological change*” OR “psychosocial change*” OR “personal change*” OR “psychological adaptation” OR “psychological adjustment” OR “psychological impact” OR “identity dissonance” OR “neurocognitive change*” OR “neurocognitive outcome*” OR “bodily integrity” OR “graft incorporation”) OR AB (personali* OR identit* OR “self concept” OR “self-concept” OR “self identity” OR “self-identity” OR “sense of self” OR selfhood OR “body image” OR embodiment OR embodied OR existential* OR “personality change*” OR “identity change*” OR “behavioral change*” OR “behavioural change*” OR “emotional change*” OR “psychological change*” OR “psychosocial change*” OR “personal change*” OR “psychological adaptation” OR “psychological adjustment” OR “psychological impact” OR “identity dissonance” OR “neurocognitive change*” OR “neurocognitive outcome*” OR “bodily integrity” OR “graft incorporation”)	322,092
3	TI (“cellular memory” OR “memory transfer” OR “memory-behavioral transfer” OR “memory behavioural transfer” OR “personality transfer” OR “behavioral transfer” OR “behavioural transfer” OR “donor personality” OR “recipient personality” OR “donor characteristic*” OR “donor trait*” OR “food preference*” OR craving* OR dream* OR (donor* N3 personali*) OR (donor* N3 identit*) OR (donor* N3 memor*) OR (donor* N3 preference*) OR (donor* N3 behavio*) OR (donor* N3 emotion*) OR (donor* N3 temperament*)) OR AB (“cellular memory” OR “memory transfer” OR “memory-behavioral transfer” OR “memory behavioural transfer” OR “personality transfer” OR “behavioral transfer” OR “behavioural transfer” OR “donor personality” OR “recipient personality” OR “donor characteristic*” OR “donor trait*” OR “food preference*” OR craving* OR dream* OR (donor* N3 personali*) OR (donor* N3 identit*) OR (donor* N3 memor*) OR (donor* N3 preference*) OR (donor* N3 behavio*) OR (donor* N3 emotion*) OR (donor* N3 temperament*))	13,752
4	S2 OR S3	333,600
5	S1 AND S4	176
**ID**	**Search Strategy for Scopus (Search Conducted on 10 August 2026)**	**Results**
1	TITLE-ABS-KEY (“heart transplant*” OR “cardiac transplant*” OR “heart transplant recipient*” OR “cardiac transplant recipient*” OR “heart recipient*” OR “cardiac recipient*” OR “transplanted heart*”)	73,942
2	TITLE-ABS-KEY (personalit* OR identit* OR “self concept” OR “self-concept” OR “self identity” OR “self-identity” OR “sense of self” OR selfhood OR “body image” OR embodiment OR embodied OR existential* OR perception* OR “personality change*” OR “identity change*” OR “behavioral change*” OR “behavioural change*” OR “emotional change*” OR “psychological change*” OR “psychosocial change*” OR “personal change*” OR “life change*” OR “psychological adaptation” OR “psychological adjustment” OR “psychological impact” OR “identity dissonance” OR “neurocognitive change*” OR “neurocognitive outcome*” OR “bodily integrity” OR “graft incorporation” OR emotion* OR temperament* OR preference* OR “food preference*” OR craving* OR memor*)	5,737,885
3	TITLE-ABS-KEY (“cellular memory” OR “memory transfer” OR “memory-behavioral transfer” OR “memory behavioural transfer” OR “personality transfer” OR “behavioral transfer” OR “behavioural transfer” OR “donor personality” OR “recipient personality” OR “donor characteristic*” OR “donor trait*” OR “food preference*” OR craving* OR dream* OR (donor* W/3 personalit*) OR (donor* W/3 identit*) OR (donor* W/3 memor*) OR (donor* W/3 preference*) OR (donor* W/3 behavio*) OR (donor* W/3 emotion*) OR (donor* W/3 temperament*))	137,382
4	(TITLE-ABS-KEY (personalit* OR identit* OR “self concept” OR “self-concept” OR “self identity” OR “self-identity” OR “sense of self” OR selfhood OR “body image” OR embodiment OR embodied OR existential* OR perception* OR “personality change*” OR “identity change*” OR “behavioral change*” OR “behavioural change*” OR “emotional change*” OR “psychological change*” OR “psychosocial change*” OR “personal change*” OR “life change*” OR “psychological adaptation” OR “psychological adjustment” OR “psychological impact” OR “identity dissonance” OR “neurocognitive change*” OR “neurocognitive outcome*” OR “bodily integrity” OR “graft incorporation” OR emotion* OR temperament* OR preference* OR “food preference*” OR craving* OR memor*)) OR (TITLE-ABS-KEY (“cellular memory” OR “memory transfer” OR “memory-behavioral transfer” OR “memory behavioural transfer” OR “personality transfer” OR “behavioral transfer” OR “behavioural transfer” OR “donor personality” OR “recipient personality” OR “donor characteristic*” OR “donor trait*” OR “food preference*” OR craving* OR dream* OR (donor* W/3 personalit*) OR (donor* W/3 identit*) OR (donor* W/3 memor*) OR (donor* W/3 preference*) OR (donor* W/3 behavio*) OR (donor* W/3 emotion*) OR (donor* W/3 temperament*)))	5,804,732
5	(TITLE-ABS-KEY (“heart transplant*” OR “cardiac transplant*” OR “heart transplant recipient*” OR “cardiac transplant recipient*” OR “heart recipient*” OR “cardiac recipient*” OR “transplanted heart*”)) AND ((TITLE-ABS-KEY (personalit* OR identit* OR “self concept” OR “self-concept” OR “self identity” OR “self-identity” OR “sense of self” OR selfhood OR “body image” OR embodiment OR embodied OR existential* OR perception* OR “personality change*” OR “identity change*” OR “behavioral change*” OR “behavioural change*” OR “emotional change*” OR “psychological change*” OR “psychosocial change*” OR “personal change*” OR “life change*” OR “psychological adaptation” OR “psychological adjustment” OR “psychological impact” OR “identity dissonance” OR “neurocognitive change*” OR “neurocognitive outcome*” OR “bodily integrity” OR “graft incorporation” OR emotion* OR temperament* OR preference* OR “food preference*” OR craving* OR memor*)) OR (TITLE-ABS-KEY (“cellular memory” OR “memory transfer” OR “memory-behavioral transfer” OR “memory behavioural transfer” OR “personality transfer” OR “behavioral transfer” OR “behavioural transfer” OR “donor personality” OR “recipient personality” OR “donor characteristic*” OR “donor trait*” OR “food preference*” OR craving* OR dream* OR (donor* W/3 personalit*) OR (donor* W/3 identit*) OR (donor* W/3 memor*) OR (donor* W/3 preference*) OR (donor* W/3 behavio*) OR (donor* W/3 emotion*) OR (donor* W/3 temperament*))))	1847
**ID**	**Search Strategy for Web of Science (Search Conducted on 10 August 2026)**	**Results**
1	TS = (“heart transplant*” OR “cardiac transplant*” OR “heart transplant recipient*” OR “cardiac transplant recipient*” OR “heart recipient*” OR “cardiac recipient*” OR “transplanted heart*”)	55,214
2	TS = (personalit* OR identit* OR “self concept” OR “self-concept” OR “self identity” OR “self-identity” OR “sense of self” OR selfhood OR “body image” OR embodiment OR embodied OR existential* OR “personality change*” OR “identity change*” OR “behavioral change*” OR “behavioural change*” OR “emotional change*” OR “psychological change*” OR “psychosocial change*” OR “personal change*” OR “life change*” OR “psychological adaptation” OR “psychological adjustment” OR “psychological impact” OR “identity dissonance” OR “neurocognitive change*” OR “neurocognitive outcome*” OR “bodily integrity” OR “graft incorporation”)	1,186,034
3	TS = (“cellular memory” OR “memory transfer” OR “memory-behavioral transfer” OR “memory behavioural transfer” OR “personality transfer” OR “behavioral transfer” OR “behavioural transfer” OR “donor personality” OR “recipient personality” OR “donor characteristic*” OR “donor trait*” OR “food preference*” OR craving* OR dream* OR (donor* NEAR/3 personalit*) OR (donor* NEAR/3 identit*) OR (donor* NEAR/3 memor*) OR (donor* NEAR/3 preference*) OR (donor* NEAR/3 behavio*) OR (donor* NEAR/3 emotion*) OR (donor* NEAR/3 temperament*))	88,480
4	#2 OR #3	1,268,561
5	#1 AND #4	401
**ID**	**Search Strategy for PsychINFO via EBSCOhost (Search Conducted on 10 August 2026)**	**Results**
1	(DE “Organ Transplantation” AND (TI (heart OR cardiac) OR AB (heart OR cardiac))) OR TI (“heart transplant*” OR “cardiac transplant*” OR “heart transplant recipient*” OR “cardiac transplant recipient*” OR “heart recipient*” OR “cardiac recipient*” OR “transplanted heart*”) OR AB (“heart transplant*” OR “cardiac transplant*” OR “heart transplant recipient*” OR “cardiac transplant recipient*” OR “heart recipient*” OR “cardiac recipient*” OR “transplanted heart*”)	719
2	DE “Personality” OR DE “Personality Change” OR DE “Personality Traits” OR DE “Self-Concept” OR DE “Emotional Adjustment” OR DE “Identity Crisis” OR DE “Emotions” OR DE “Emotional Responses” OR DE “Emotional States” OR DE “Memory” OR DE “Body Image” OR TI (personalit* OR identit* OR “self concept” OR “self-concept” OR “self identity” OR “self-identity” OR “sense of self” OR selfhood OR “body image” OR embodiment OR embodied OR existential* OR perception* OR “personality change*” OR “identity change*” OR “behavioral change*” OR “behavioural change*” OR “emotional change*” OR “psychological change*” OR “psychosocial change*” OR “personal change*” OR “life change*” OR “psychological adaptation” OR “psychological adjustment” OR “psychological impact” OR “identity dissonance” OR “neurocognitive change*” OR “neurocognitive outcome*” OR “bodily integrity” OR “graft incorporation” OR emotion* OR temperament* OR preference* OR “food preference*” OR craving* OR memor*) OR AB (personalit* OR identit* OR “self concept” OR “self-concept” OR “self identity” OR “self-identity” OR “sense of self” OR selfhood OR “body image” OR embodiment OR embodied OR existential* OR perception* OR “personality change*” OR “identity change*” OR “behavioral change*” OR “behavioural change*” OR “emotional change*” OR “psychological change*” OR “psychosocial change*” OR “personal change*” OR “life change*” OR “psychological adaptation” OR “psychological adjustment” OR “psychological impact” OR “identity dissonance” OR “neurocognitive change*” OR “neurocognitive outcome*” OR “bodily integrity” OR “graft incorporation” OR emotion* OR temperament* OR preference* OR “food preference*” OR craving* OR memor*)	1,616,102
3	TI (“cellular memory” OR “memory transfer” OR “memory-behavioral transfer” OR “memory behavioural transfer” OR “personality transfer” OR “behavioral transfer” OR “behavioural transfer” OR “donor personality” OR “recipient personality” OR “donor characteristic*” OR “donor trait*” OR “donor-recipient correspondence*” OR “donor recipient correspondence*” OR “donor-recipient similarit*” OR “donor recipient similarit*” OR “food preference*” OR craving* OR dream* OR (donor* N3 personalit*) OR (donor* N3 identit*) OR (donor* N3 memor*) OR (donor* N3 preference*) OR (donor* N3 behavio*) OR (donor* N3 emotion*) OR (donor* N3 temperament*) OR (donor* N3 trait*)) OR AB (“cellular memory” OR “memory transfer” OR “memory-behavioral transfer” OR “memory behavioural transfer” OR “personality transfer” OR “behavioral transfer” OR “behavioural transfer” OR “donor personality” OR “recipient personality” OR “donor characteristic*” OR “donor trait*” OR “donor-recipient correspondence*” OR “donor recipient correspondence*” OR “donor-recipient similarit*” OR “donor recipient similarit*” OR “food preference*” OR craving* OR dream* OR (donor* N3 personalit*) OR (donor* N3 identit*) OR (donor* N3 memor*) OR (donor* N3 preference*) OR (donor* N3 behavio*) OR (donor* N3 emotion*) OR (donor* N3 temperament*) OR (donor* N3 trait*))	40,377
4	S2 OR S3	1,633,126
5	S1 AND S4	235

**Table A2:** Proposed clinical inquiry prompts for post-heart-transplant follow-up.

Domain	Suggested Clinical Inquiry	Clinical Consideration
Emotional adjustment	“Since the transplant, have you noticed any changes in your mood, emotional responses, anxiety, or sense of psychological well-being?”	Explore persistence, severity, distress, and impact on daily functioning; consider formal psychological assessment when indicated.
Identity and self-concept	“Has the transplant changed the way you think about yourself, your identity, or who you are?”	Assess difficulties in self-continuity, identity integration, or adjustment to the post-transplant self.
Graft embodiment	“How do you experience the transplanted heart as part of your body?”	Explore feelings of integration, foreignness, bodily unfamiliarity, or altered body perception.
Changes in preferences or behavior	“Have you noticed any unexpected changes in your preferences, habits, interests, or behavior since transplantation?”	Clarify onset, context, persistence, perceived significance, and possible alternative explanations, including medication, recovery, and lifestyle changes.
Donor-related thoughts and perceived correspondences	“Do you ever connect any changes or experiences you have noticed with the donor or with what you know about the donor?”	Explore the recipient’s interpretation without confirming or dismissing donor-related causal explanations; document prior exposure to donor information where relevant.
Dreams, sensory, or memory-like experiences	“Have you experienced unusual dreams, sensations, images, memories, or other experiences that you associate with the transplant?”	Assess frequency, distress, meaning, functional impact, and whether further psychological or clinical evaluation is required.
Guilt, indebtedness, and existential concerns	“Do you experience feelings of guilt, indebtedness, responsibility toward the donor, or difficulty reconciling your survival with the donor’s death?”	Explore existential distress, complicated meaning-making, and need for psychological, social, or spiritual support.
Social, relational, and spiritual adjustment	“Has transplantation changed your relationships, social roles, values, spirituality, or priorities in life?”	Identify changes affecting relationships, social participation, coping, or existential well-being.
Impact on functioning and treatment	“Do any of these changes or experiences interfere with your daily life, relationships, treatment, or ability to follow your post-transplant care?”	Clinically significant distress, impairment, or effects on adherence should prompt further multidisciplinary assessment.

Note: These questions are intended as prompts to support person-centered clinical inquiry during post-heart-transplant follow-up. They were derived from the experiential domains identified in this review and do not constitute a validated screening instrument, diagnostic tool, or standardized clinical protocol. Responses should be interpreted within the recipient’s broader psychological, medical, relational, cultural, and spiritual context. Where clinically significant distress, functional impairment, or concerns regarding treatment adherence are identified, appropriate multidisciplinary assessment or referral should be considered.
